# Caregivers’ Willingness to Vaccinate Their Children Against COVID-19 in Saudi Arabia: A Cross-Sectional Survey

**DOI:** 10.7759/cureus.17243

**Published:** 2021-08-17

**Authors:** Zainab Almusbah, Zahraa Alhajji, Zahraa Alshayeb, Rania Alhabdan, Sukainah Alghafli, Mohammed Almusabah, Fatimah Almuqarrab, Isra Aljazeeri, Fida Almuhawas

**Affiliations:** 1 College of Medicine, King Faisal University, Al-Ahsa, SAU; 2 General Surgery, King Fahad General Hospital, Al-Ahsa, SAU; 3 Dermatology, King Fahad General Hospital, Al-Ahsa, SAU; 4 Otology, King Abdullah Ear Specialist Center (KAESC) College of Medicine, King Saud University Medical City (KSUMC) King Saud University, Riyadh, SAU; 5 Otorhinolaryngology – Head & Neck, King Abdullah Ear Specialist Center (KAESC) King Abdulaziz University Hospital, King Saud University, Riyadh, SAU

**Keywords:** covid-19, coronavirus, vaccines, pediatrics, saudi arabia

## Abstract

Background

In early 2021, vaccination against COVID-19 became one of the most important measures needed to control the pandemic.

Objectives

This study aimed to investigate the levels of acceptance and factors affecting the decisions among Saudi parents and caregivers of children under 12 for getting them vaccinated.

Design and setting

A cross-sectional survey of 1000 caregivers and parents was carried out from May 2021 to June 2021. The participants were from the eastern, central, southern, western, and northern provinces of Saudi Arabia.

Materials and methods

The data were collected and managed using Microsoft Excel (Microsoft^®^ Corp., Redmond, WA) and analyzed using SPSS version 23 (IBM Corp., Armonk, NY).

Results

Of the 1000 respondents, 281 (28.1%) reported they would vaccinate their children, 346 (34.6%) rejected vaccination, and 373 (37.3%) were not sure. Most caregivers (24.1%; n = 241) reported protecting children as the principal reason for accepting vaccination. Regarding those rejecting the vaccine, the most common concern was that children might experience side effects (42.9%; n = 429).

Limitations

The acceptance of the COVID-19 vaccine is dynamic and changes with legislations and public awareness policies.

Conclusions

COVID-19 vaccine acceptance for children was low at the time of this study compared to the United Kingdom and United States. To achieve vaccination coverage that is adequate for herd immunity in Saudi Arabia, intense educational and awareness strategies are needed.

## Introduction

COVID-19 was first reported to the WHO on December 31, 2019, in Wuhan City, Hubei Province, China. On March 11, 2020, the WHO declared COVID-19 as a global pandemic [1,2]. Since then, protective measures have been instituted worldwide to control the spread of the disease. For any infectious disease, the availability of safe and effective vaccines is important for establishing herd immunity and preventing infection spread [3]. At the time of writing this paper, at least seven vaccines were approved by most countries. Over 200 candidate vaccines are at some stage of development, and at least 60 of these are at the clinical development stage [4]. Vaccine candidates are targeted for use in outbreak settings and provide long-term protection for those at risk of COVID-19, such as health care workers [5].

During a pandemic, conventional vaccine approval processes may be waived. For instance, some vaccine candidates have been granted fast-track licensure by the US Food and Drug Administration [6]. Even when safe and effective vaccines have been developed, a major barrier to reaching herd immunity is community acceptance for getting vaccinated. Hesitancy levels and reasons vary in different communities, depending on the nature of the vaccines, health care systems, accessibility, and cultural and social factors. Evidence shows that the newer the vaccine, the higher the hesitancy level among the public, which may pose a major challenge for persuading people to get a COVID-19 vaccine [7].

Recent studies have been conducted on different populations to determine the acceptance of COVID-19 vaccines. In the United Kingdom, 55.8% of survey respondents said they would accept vaccination for themselves, and 48% would accept it for their children [8]. In the United States, 80% of Americans reported that they would accept the vaccination for themselves and their children [9]. In another international study investigating global caregivers’ intentions, 65.2% of respondents reported that they are planning to vaccinate their children [10]. According to the evidence, the most consistent reason for COVID-19 vaccine hesitancy is the novelty of the vaccines. The other two main determinants of caregivers’ hesitancy toward vaccinating children are safety concerns and the perception that children are rarely affected by COVID-19 [8-10].

In Saudi Arabia, a study conducted on the adult population demonstrated a 64.7% acceptance of a hypothetical vaccine. Participants’ trust in the health system and the perceived risk of getting infected were two major determining factors for this willingness [11]. However, the willingness of caregivers to get their children vaccinated and the determinants of vaccine hesitancy need to be investigated to plan a vaccination program once vaccines are available. To the best of our knowledge, this study is the first of its kind in Saudi Arabia.

## Materials and methods

Study design and setting

The present study was a cross-sectional survey of the Saudi population from the eastern, central, southern, western, and northern provinces. It investigated the factors that affect parents’ and family caregivers’ acceptance of COVID-19 vaccines for their children. This study was approved by the research ethics committee of the University Hospital (approval number E-20-5558). After the participation details were explained, informed consent was provided by all participants before proceeding to the survey link.

The study was carried out from May 12th, 2021 to June 28th, 2021. Eligible participants were Saudi parents and caregivers of one child or more aged up to 12 years; participants who did not complete the entire survey were excluded. The survey was designed using Google Forms, and the data were collected in an encrypted Google drive. Distribution was via a link on social media channels (Twitter, WhatsApp, and Telegram). The respondents were asked for their socio-demographic characteristics, the health status of their children, commitment to the national vaccination program, and their beliefs about COVID-19 vaccines. The second part of the survey evaluated their level of vaccine hesitancy and the reasons for their decisions regarding vaccination.

Survey design and pre-testing

The survey items were extracted from a similar study conducted with a British population [8]. The survey was translated by two bilingual health-science researchers and reviewed by two other bilingual researchers. The translated survey was then distributed among 10 randomly chosen participants to examine its comprehensibility, time taken, and any technical problems with the survey link. The reviewed survey was then distributed to the target population.

Analysis

The data were collected and managed using Microsoft Excel version 16.3 (Microsoft® Corp., Redmond, WA) and analyzed using SPSS version 23 (IBM Corp., Armonk, NY). Statistical significance, confidence interval, and study power were set at p < 0.05, 95%, and 80%, respectively. Percentage was used for the descriptive values. Chi-square was used to evaluate the differences between categorical variables, and a paired t-test was used to compare the willingness to vaccinate oneself and willingness to vaccinate their children.

## Results

The study included 1000 parents and caregivers (Table 1). Most survey participants lived in the eastern provinces (64.1%; n = 641), followed by the central (17.8%; n = 178), western (8.9%; n = 89), southern (8.4%; n = 84), and northern (5%; n = 5) provinces. Of 1000 parents and caregivers, 788 (78.8%) were female, and most were the children’s mothers (65.8%; n = 658). This gender imbalance in our study sample likely affected the robustness of the study’s findings, since most of the reported vaccine rejections were from female caregivers. Most children had received the vaccinations appropriate for their age, as per the national immunization program (78.9%; n = 789). Regarding the medical histories of the children, most (92.8%; n = 928) did not have any chronic diseases. Furthermore, 960 (96.9%) children did not use regular medication, and most children had not had COVID-19 (89.4%; n = 894). Only 243 (24.3%) of the parents and caregivers had been vaccinated against seasonal flu in the current year, and 476 (47.6%) had never been vaccinated against seasonal flu (Table 2).

**Table 1:** Characteristics of the study population.

Characteristics		Frequency (%)
Residence	Eastern province	641 (64.1%)
	Central province	178 (17.8%)
	Western province	89 (8.9%)
	South province	84 (8.4%)
	North province	5 (0.5%)
Relationship with child	Mother	658 (65.8%)
	Father	194 (19.4%)
	Sibling	76 (7.6%)
	Aunt/uncle	60 (6%)
	Grandparents	12 (1.2%)
Gender of caregiver	Female	788 (78.8%)
	Male	212 (21.2%)
Age of child	Newborn to 30 days	14 (1.4%)
	One month to 2 years	232 (23.2%)
	2 years to 6 years	402 (40.2%)
	6 years to 12 years	352 (35.2%)
Gender of child	Female	470 (47%)
	Male	530 (53%)
Chronic diseases	Yes	72 (7.2%)
	No	928 (92.8%)
Medications	Yes	40 (4%)
	No	960 (96%)
Child has received age-appropriate vaccinations	Yes	786 (78.6%)
	No	117 (11.7%)
	Not sure	97 (9.7%)
Caregiver has received seasonal flu vaccine this year	Yes	243 (24.3%)
	No	757 (75.7%)
Caregiver has ever received seasonal flu vaccine	Yes	476 (47.6%)
	No	524 (52.4%)
Child ever infected with COVID-19	Yes	106 (10.6%)
	No	894 (89.4%)

**Table 2:** Variables and categories among the study population.

Variables	Are you willing to get the COVID-19 vaccine if approved by the Saudi FDA?					Are you willing to get the COVID-19 vaccine for your child if approved by the Saudi FDA?		
	Yes		Pearson Chi-Square (2-sided significance)		95% CI	Yes	Pearson Chi-Square (2-sided significance)	95% CI
									Residence								
Eastern province	26.80%		11.77 (p = 0.15)		0.12-0.18	25.30%	3.04 (p = 0.93)	0.91-0.95
Central province	33.70%					28.10%		
Western province	29.20%					27%		
South province	23.80%					20.20%		
North province	37.50%					37.50%		
Relationship with child								
Mother	23.90%		22.55 (p = 0.005)		0.00-0.01	21.30%	20.7 (p = 0.006)	0.00-0.01
Father	37.10%					35.60%		
Sibling	32.90%					30.30%		
Aunt/Uncle	38.30%					33.30%		
Grandparents	33.30%					33.30%		
Gender of caregiver								
Female	25.90%		10.18 (p = 0.007)		0.00-0.01	23%	13.54 (p = 0.001)	0.00-0.004
Male	36.30%					35.40%		
Age of child								
Newborn to 1 month						21.40%	2.88 (p = 0.82)	0.79-0.85
1 month to 2 years						25.9%		
2-6 years old						24.40%		
6-12 years old						27%		
Chronic Disease								
Present						25.80%	1.23 (p = 0.52)	0.48-0.56
Absent						23.60%		
Medication								
Present						25.10%	3.44 (P = 0.18)	0.15
Absent						37.50%		0.21
Child has received age-appropriate vaccinations								
No		28.20%				25.60%	3.88 (P = 0.43)	0.39
Yes		28.90%				26.50%		0.47
Caregiver has received seasonal flu vaccination this year								
No		23.10%				20.90%	40.85 (P = 0.00)	0
	Yes			43.60%							40.30%	0.005
Caregiver has received seasonal flu vaccination ever								
No		22.90%				21%	12.78 (P = 0.002)	0
Yes		33.80%				30.70%		0.005
Child ever infected with COVID-19								
No						25.70%	0.51 (P = 0.78)	0.74
Yes						24.50%		0.81
Social distancing								
Do not believe		31.60%				29.80%	0.9 (P = 0.62)	0.58
Believe		27.90%				25.40%		0.66
Washing hands								
Do not believe		35.90%				30.80%	0.84 (P = 0.62)	0.58
Believe		27.80%				25.40%		0.66
Wearing mask								
Do not believe		30.20%				27%	0.37 (P = 0.83)	0.8
Believe		28%				25.50%		0.86

**Figure 1:**
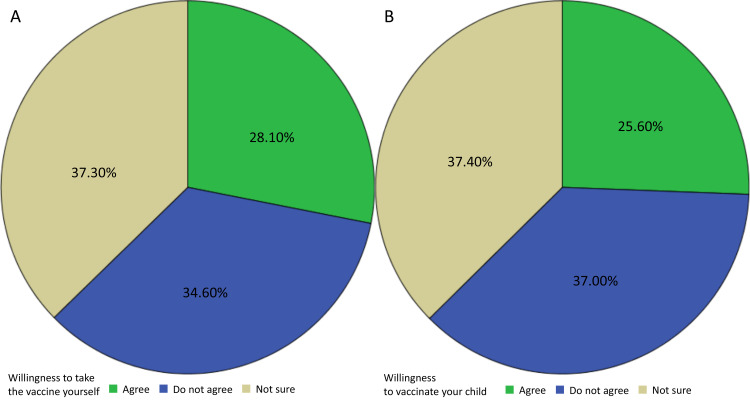
(A) Caregivers’ willingness to be vaccinated; (B) Caregivers’ willingness to vaccinate their children.

Of the 1000 respondents, 281 (28.1%) said that they would get vaccinated, 346 (34.6%) rejected vaccination, and 373 (37.3%) reported they were not yet sure of their decision. Additionally, 256 (25.6%) agreed to get their child vaccinated, 370 (37%) rejected vaccination of their child, and 374 (37.4%) reported they were not yet sure of their decision (Figure 1).

**Figure 2:**
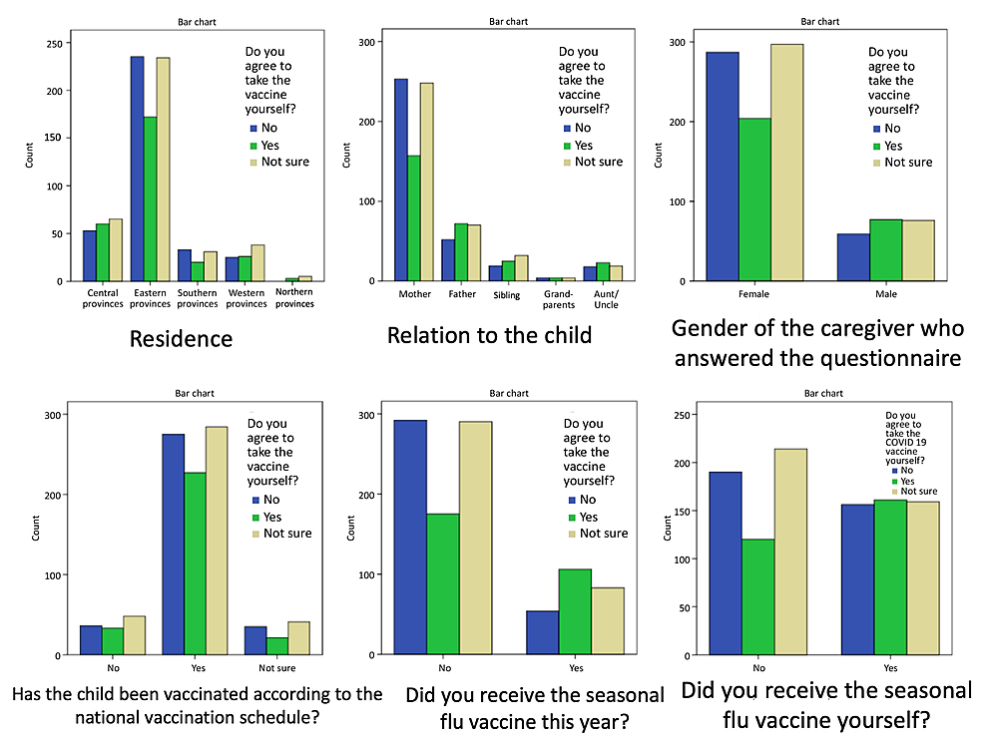
Caregivers’ willingness to be vaccinated in relation to their demographic information.

**Figure 3:**
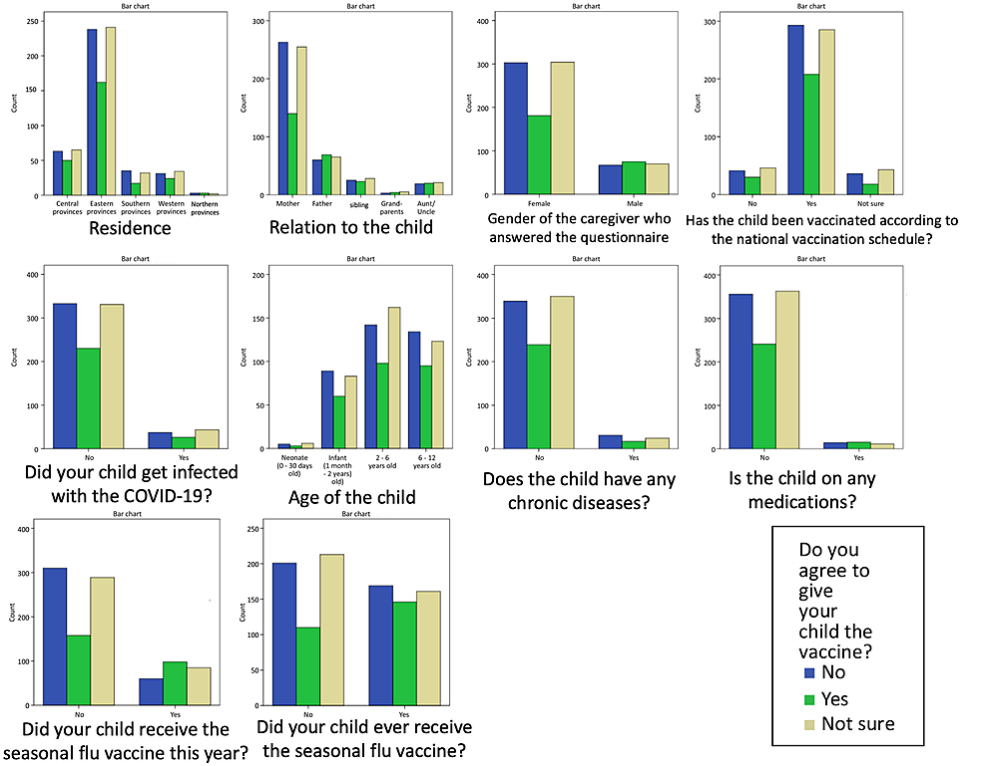
Caregivers’ willingness to vaccinate their children in relation to their demographic information.

No differences were found in vaccine acceptance for themselves or the children among the different provinces (chi-square p-values 0.15 and 0.93, respectively). Mothers’ and females’ acceptance rates (23.9% and 25.9%, respectively) were the lowest for accepting a COVID-19 vaccine for themselves or their children. Those who received the seasonal flu vaccine in the current year or had ever received it had significantly higher acceptance rates for getting vaccinated (p < 0.001) (Figures 2, 3).

The acceptance rate for vaccinating the child was not significantly different among different age groups (chi-square p-value 0.82). Whether the child had any chronic diseases, took prescribed medications, had up-to-date vaccinations, or had been infected with COVID-19, had no significant effects on the decision to vaccinate the child (p = 0.52, 0.18, and 0.43, respectively). Most of the survey participants had a good awareness of COVID-19 prevention methods. Social distancing, handwashing, and wearing masks were believed to be important protective measures by 94.2%, 96%, and 93.6% of the participants, respectively. Despite their beliefs in these preventative measures, the rate of acceptance for oneself or their child getting vaccinated was not significantly different from those participants who did not believe in the preventative measures (p = 0.17 to 0.9).

**Figure 4:**
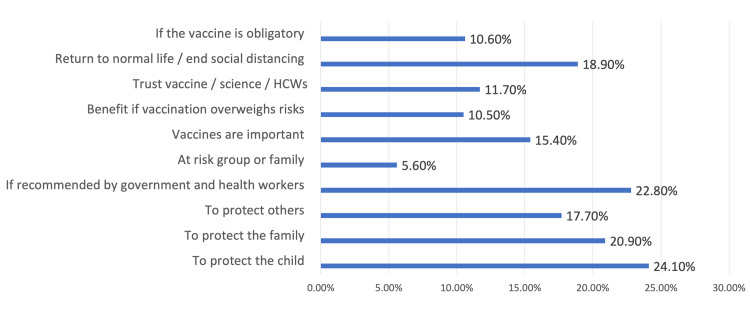
Caregivers’ reasons for vaccinating their children against COVID-19. HCWs: Health care workers

**Figure 5:**
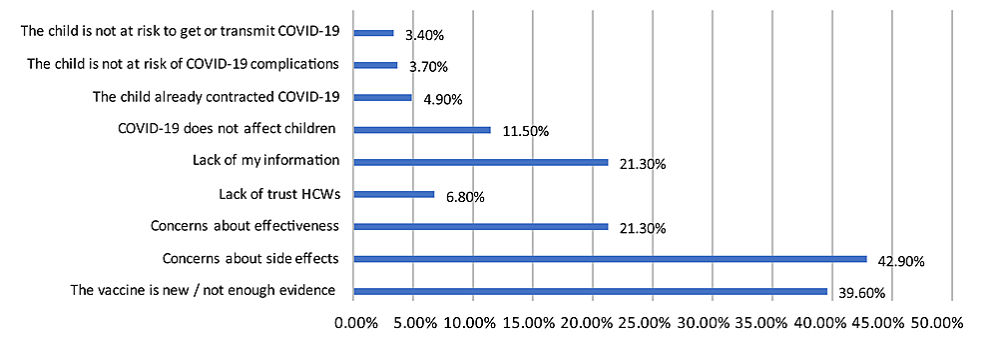
Caregivers’ reasons for not vaccinating their children against COVID-19. HCWs: Health care workers

Protection of the child was the principal reason that most caregivers accepted vaccination (24.1%; n = 241). On the other hand, the most common concern expressed by survey participants who rejected vaccination was the side effects of the vaccine (42.9%; n = 429) (Figures 4, 5).

## Discussion

Vaccinations have some of the most remarkable public health benefits. In the case of COVID-19, it may be the most feasible way to end the pandemic and reach herd immunity [3]. However, vaccine hesitancy is a major challenge when bringing vaccination programs into communities. In this cross-sectional study, COVID-19 vaccine hesitancy and acceptance were investigated, and our study results skewed toward rejecting vaccination. Our findings demonstrate that around 25% of respondents were willing to vaccinate their children, which is in contrast to American and British surveys, which found that 80% and 89% of parents were willing to vaccinate their children, respectively [8-10].

Additionally, in contrast to the previously reported independent factors [10], child’s age, being up-to-date on vaccinations, and the child’s medical and medication histories, were not found to significantly affect participants’ willingness to vaccinate their children. In parallel with Goldman et al.’s study on H1N1 vaccination willingness [12], our data demonstrate a preferential acceptance rate among male compared to female parents and caregivers. A protective behavioral variation between fathers and mothers is suggested by Goldman et al. and Brussoni and Olsen [10,13]. Furthermore, the vaccination willingness rate was higher in our study if the child or their caregiver was vaccinated against seasonal influenza in the last year, and this finding was statistically significant.

In parallel with studies by Bell et al. and Goldman et al. [8,10], protection of the child was the number one motivation for accepting vaccination, and fears of a vaccine’s side effects along with its novelty were the two main reasons for rejecting vaccination. The next most common reason for accepting vaccination was protecting others and returning to normal life. The Ministry of Health in Saudi Arabia has provided scientifically based health information through their official website [14], and there have been local restrictions prohibiting misinformation on social media. A lack of trust in the healthcare system only represents a minor concern for Saudis, and the majority of those who accept vaccination for their children would do so if the health authorities recommended it, and this reflects their trust.

The participants were asked about their knowledge of COVID-19 protective measures; however, we failed to identify any relationship between parents’ or caregivers’ awareness and hesitancy toward vaccination. Our findings provide foundational data for future vaccination campaigns that address parents’ and caregivers’ concerns and misconceptions and provide up-to-date evidence-based information on the perceived risks of contracting COVID-19 and vaccinations for children.

This study is limited by the dynamic nature of the COVID-19 pandemic. The results of the study can only represent the population at the time that the survey was conducted. Furthermore, since there may be large differences in the impact of COVID-19 and healthcare system policies in different countries, it is not possible to generalize the results of this study to other parts of the world. Another limitation of this study is its inability to determine the extent of voluntary response bias. This voluntary response bias may have led to the predominance (78.8%) of female respondents in this study. This same issue was found in a previous study [8], where 95% of the respondents were female.

At the time of ending this questionnaire survey, there were 18 million Saudis who received at least one dose of their COVID-19 vaccination [15]. With a reported Saudi population of around 32.6 million adults in 2021, the proportion of vaccinated adults reaches up to 55% [16]. This might indicate the presence of a voluntary response bias, in which those who did not approve of vaccination were more motivated to complete the survey. The response rate could not be obtained due to the method of questionnaire disturbance.

## Conclusions

The willingness of parents and caregivers to vaccinate their children was low among the Saudi population at the time of this study. Concerns regarding the potential side effects and safety of the vaccines were the most common barriers to the willingness to vaccinate children. In contrast, protecting their children was the most common motivation for the uptake of vaccination. This study highlights the magnitude of the hesitancy of Saudi parents and caregivers for vaccinating their children. When the Saudi FDA grants approval for vaccinating children against COVID-19, a mass public education program is warranted to ensure acceptable nationwide vaccination rates. A structured awareness strategy will be needed to alleviate the fears and concerns of parents and caregivers.
